# Supplementary material for: COVID-19 is linked to changes in the time–space dimension of human mobility
Source: Nat Hum Behav. 2023 Jul 27;7(10):1729–39. doi: 10.1038/s41562-023-01660-3 (PMC10593607; doi:10.1038/s41562-023-01660-3)
Supplement: Supplementary file 1 — Supplementary Figs. 1–9, discussion and Table 1. [file 41562_2023_1660_MOESM1_ESM.pdf]

nature human behaviour

Article

https://doi.org/10.1038/s41562-023-01660-3

COVID-19 is linked to changes in the time–space dimension of human mobility

In the format provided by the authors and unedited

S1 Data Information

the content of the datasets used in this work that contains information about the radius of gyration, out-of-home trips, trips to green areas, and duration of the trips is described as follows.

S1.1 Radius of Gyration

Description of the data used to analyse the spatial dimension of mobility, i.e. radius of gyration. Each item corresponds to a column in the dataset.

year: Year when the location information was saved in the database.

week: Week number according to the ISO-8601 standard where weeks start on Monday. It is a number between 1 and 52 (or 53 for leap years).

radius: The average radius of gyration in kilometres for the users inside a region at a given week of the year. The values were normalised using a min-max normalisation to comply with data sharing policies.

pings: Represents the number of pigs (sync connections sent from the users' devices to the data server). The values were also normalised using a min-max normalisation to comply with data sharing policies.

geo_code: Unique codes for local authority districts (LAD) and unitary authorities (UA) in the United Kingdom.

S1.2 Out-of-home Trips

Description of the data used to analyse the temporal dimension of mobility, i.e. mobility synchronisation. Each item corresponds to a column in the dataset.

year: Year when the location information was saved in the database.

day: Number between 1 and 365 (or 366 for leap years) where January 1 is day 1. This indicates the day when the out-of-home event was identified.

hour: Integer between 0 and 24 which represents the hour when the Out-of-Home event was identified.

trips: Total number of times that the users in a given area left home at a given period of time, i.e., the number of Out-of-home trips. The values were normalised using a min-max normalisation to comply with data sharing policies.

geo_code: Unique codes for local authority districts (LAD) and unitary authorities (UA) in the United Kingdom.

S1.3 Trips to Green Areas

Description of the data used to analyse the visits to green ares. Each item corresponds to a column in the dataset.

year: Year when the location information was saved in the database.

week: Week number according to the ISO-8601 standard where weeks start on Monday. It is a number between 1 and 52 (or 53 for leap years).

visits: Total number of users that presented a trip that contains a green area in the period considered. The values were normalised using a min-max normalisation to comply with data sharing policies.

geo_code: Unique codes for local authority districts (LAD) and unitary authorities (UA) in the United Kingdom.

S1.4 Duration of the Trips

Description of the data used to analyse the duration of the trips. Each item corresponds to a column in the dataset.

year: Year when the location information was saved in the database.

week: Week number according to the ISO-8601 standard where weeks start on Monday. It is a number between 1 and 52 (or 53 for leap years).

hour_leave: Integer between 0 and 24 which represents the hour when the user left their home geofencing area.

hour_return: Integer between 0 and 24 which represents the hour when the user first entered in their home geofencing area.

trips: Total number of users that presented that trip. The values were normalised using a min-max normalisation to comply with data sharing policies.

geo_code: Unique codes for local authority districts (LAD) and unitary authorities (UA) in the United Kingdom.

In Supplementary Figure 1 each point represents a local authority colour-coded by their country, and the axis represents the number of users and the population as a percentage of the total. A strong positive correlation between the populations compared is observed in Supplementary Figure 1 with r^2 value of 0.78. Performing the same analysis

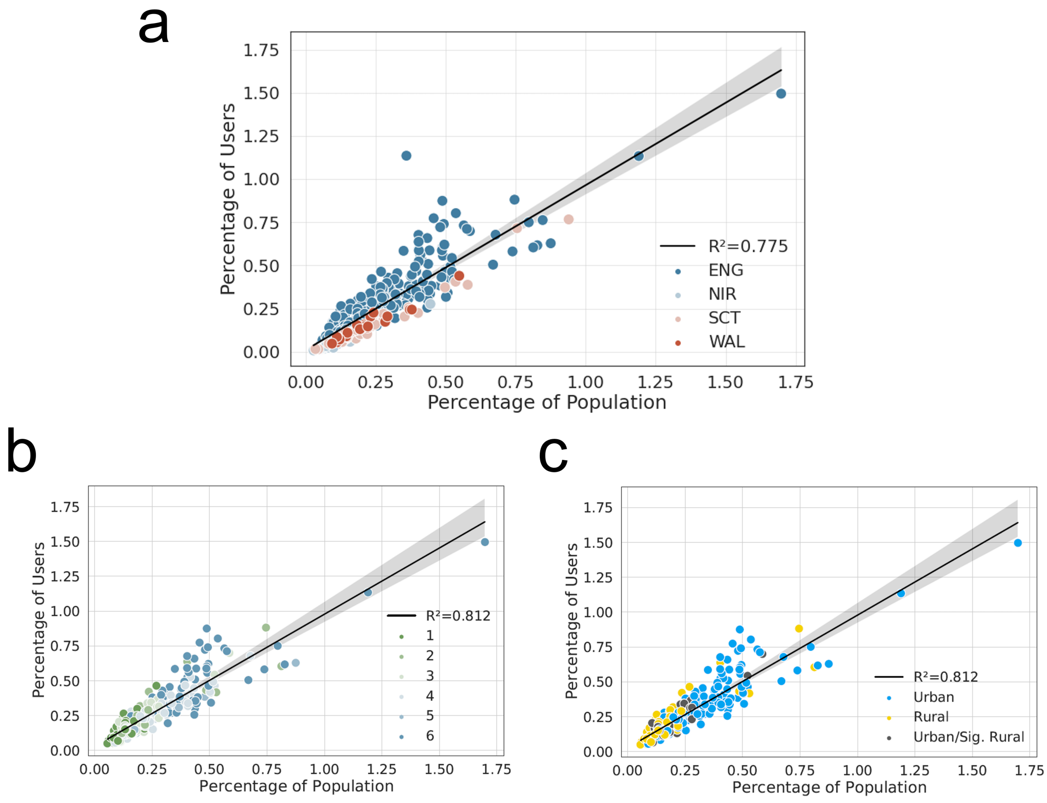

Supplementary Figure 1: Correlation between the percentage of users and the population. (a) Each point in the scatter plot represents a local authority in the UK ($N = 404$ local authorities), and the colour indicates its country. For (b) and (c), each point in the scatter plot represents a local authority in England ($N = 316$ local authorities), and the colour indicates its level of urbanisation (b) or urban-rural group (c). The x-axis represents the division of the region's population by the sum of the population of all regions. Similarly, the y-axis represents the number of users in a local authority divided by the total number of users in the data. For the results reported, $P < 0.001$ and $CI = 95\%$.

for English local authorities used in studies related to the level of urbanisation, Supplementary Figure 1 (b) and (c) shows a stronger correlation of 0.81.

The dataset contains information regarding the duration of out-of-home trips for users in the UK. The data is aggregated at the local authority level, so no information that allows user identification is included. This data will be used to assess the differences in trip duration before, after and during periods when mobility restrictions were in place.

To further assess the representativeness of the data used, we analysed the data distribution by country and urbanisation level. The results depicted in Supplementary Figure 2 do not show elements of concerns of a region of the group being able to influence their result over-weighing the distributions. Note that, for the level of urbanisation and

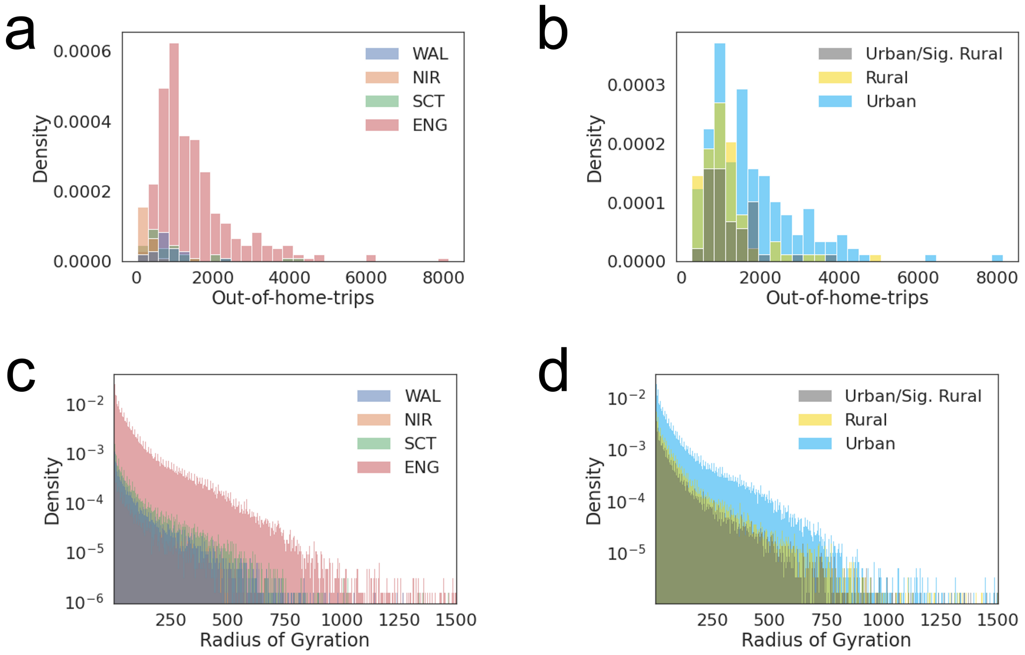

Supplementary Figure 2: Distribution of the number of out-of-home-trips by county (a) and by urban-rural group (b) and the radius of gyration (km) by county (c) and by urban-rural group (d).

socioeconomic studies, we only use data from England due to the lack of a stand data representation for all UK's countries. Moreover, before the analysis, we filter the data set and remove users with abnormal activity, such as too few logs in the server, lower location accuracy or a large range of motion (e.g. users travelling more than 100km in a day).

S2 NS-SEC Classification

The urban-rural classification adopted for England has two levels of classification. The first level classifies areas as urban, rural and urban with significant rural, while the second level classifies the areas into six levels of urbanisation ranging from level 1 to level 6. Level 1 corresponds to rural areas or low urbanisation index, while level 6 describes areas classified as major urban or high-urbanisation indexes. Supplementary Table 1 summarises the NS-SEC classes used in our analysis.

**Class**	**Description**
NS-SEC 1	Higher managerial, administrative and professional occupations
NS-SEC 2	Lower managerial, administrative and professional occupations
NS-SEC 3	Intermediate occupations (e.g. Intermediate clerical, administrative, sales, service, engineering, technical and auxiliary occupations)
NS-SEC 4	Small employers and own account workers
NS-SEC 5	Lower supervisory and technical occupations
NS-SEC 6	Semi-routine occupations
NS-SEC 7	Routine occupations
NS-SEC 8	Never worked and long-term unemployed

Supplementary Table 1: The National Statistics Socio-economic classification (NS-SEC) classes description.

S3 Spatio-temporal Metrics

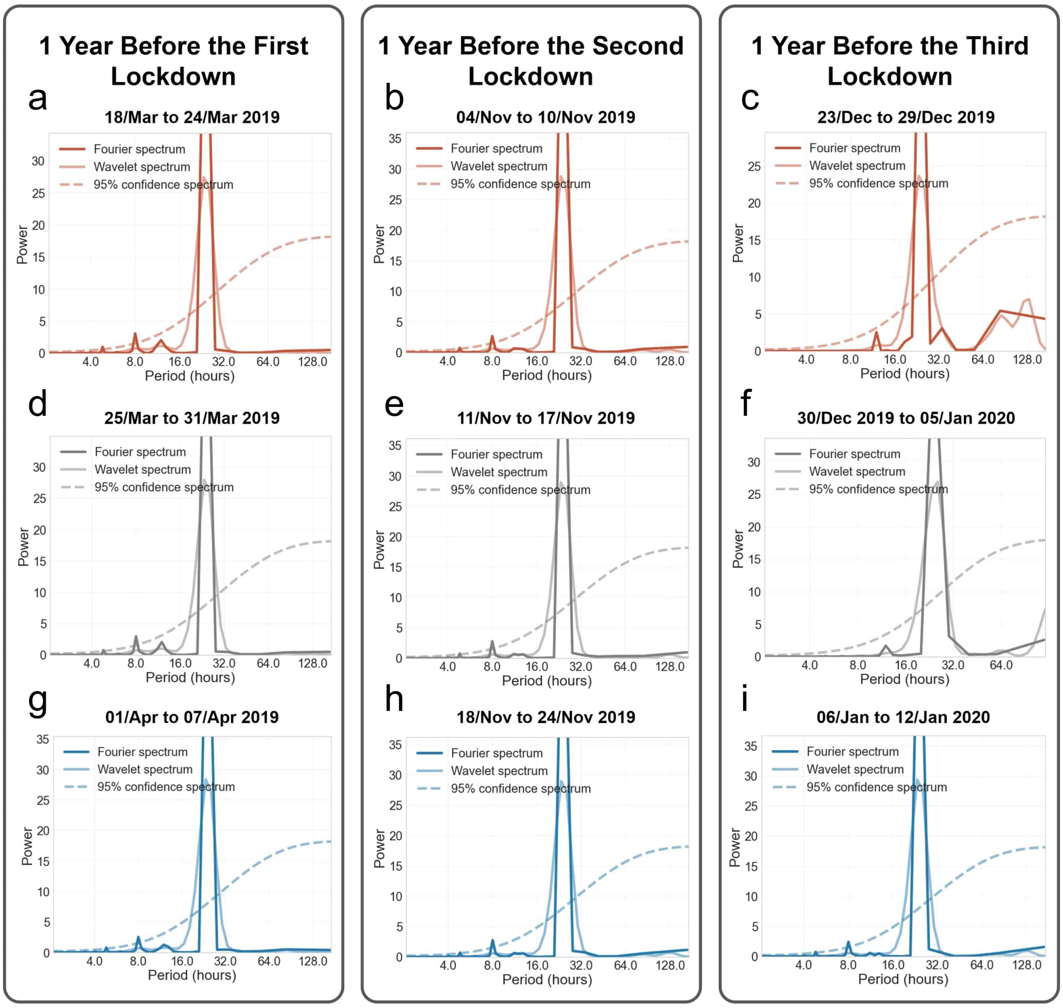

Supplementary Figure 3: Assessment of the mobility synchronisation periods for the baseline year (i.e. 2019 and January 2020). (a), (b), and (c) represent the patterns for the baseline period corresponding to the week before the announcement of the lockdowns. (d), (e), and (f), depict the baseline week's patterns when the lockdowns were announced. Lastly, (g), (h), and (i) show the baseline patterns for the week corresponding to the second week of lockdown. The synchronisation periods above the 95% confidence interval is based on the global wavelet and Fourier for the *out-of-home* trips spectra on ea, ch period. Note that, in the baseline, the most significant periods are t, o the, and 24hrs. However, since the 24hrs components dominate the other, it was not considered for calculating the mobility synchronisation metric.

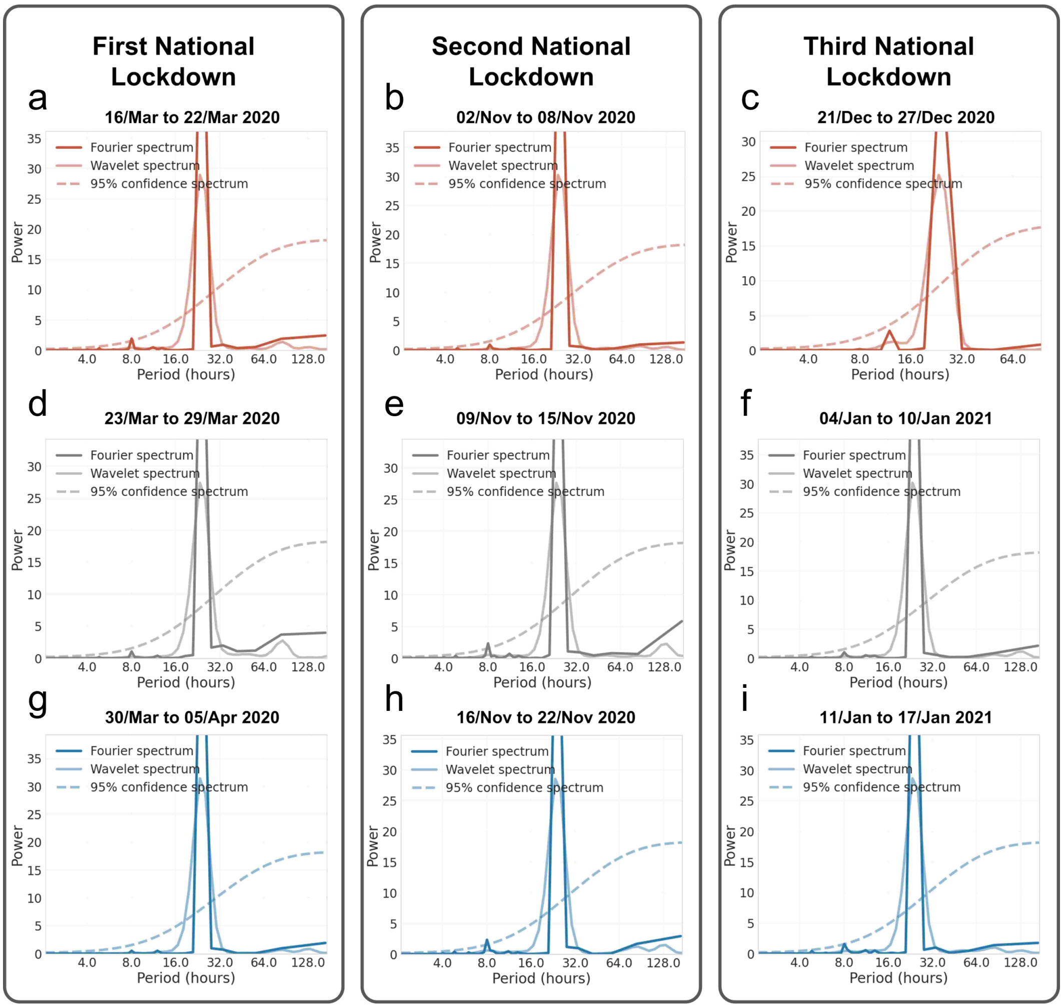

Supplementary Figure 4: Assessment of the mobility synchronisation in the UK before the national lockdowns (a), (b), and (c), in the first week of the lockdowns (d), (e), and (f), and in the second week of the lockdowns (g) and (h) The synchronisation periods above the 95% confidence interval is based on the global wavelet and Fourier for the *out-of-home* trips spectra on each period. Note that the synchronisation pattern was mostly changed during the lockdown period as (d) and (e) show a reduction in the spike at 8hrs mark when compared to the period before first lockdown (a).

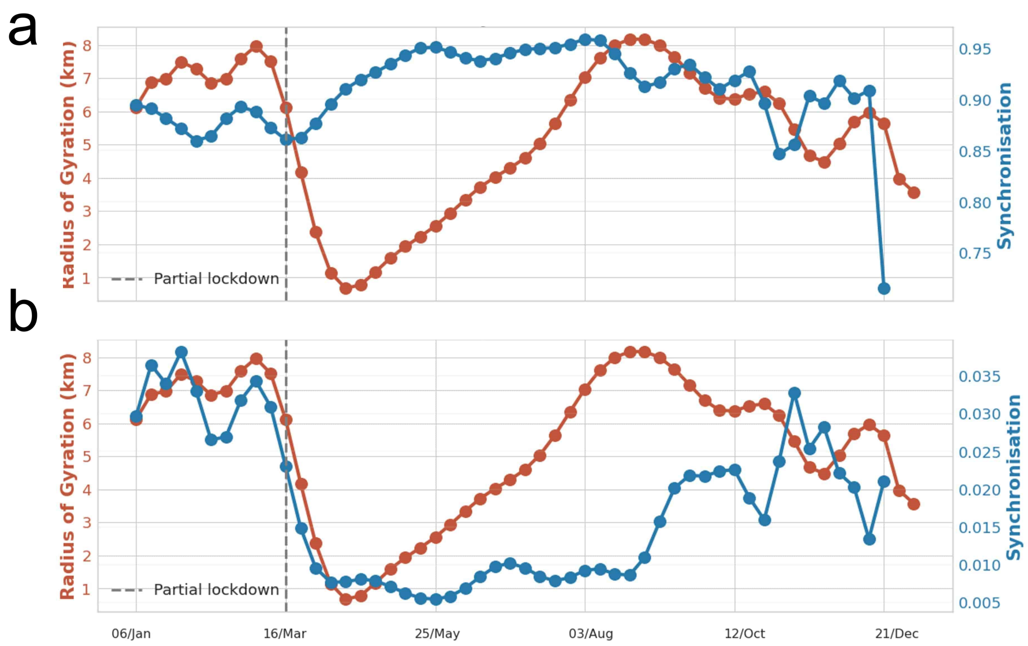

Supplementary Figure 5: Comparison between the Spatio-temporal dimensions of mobility. (a) shows the calculation of the mobility synchronisation metric using the 24hrs components, while (b) depicts the behaviour of this metric without it. Note the similarities in the trends of both metrics in (b)) for the period before the first lockdown.

S4 Mobility Patterns in England

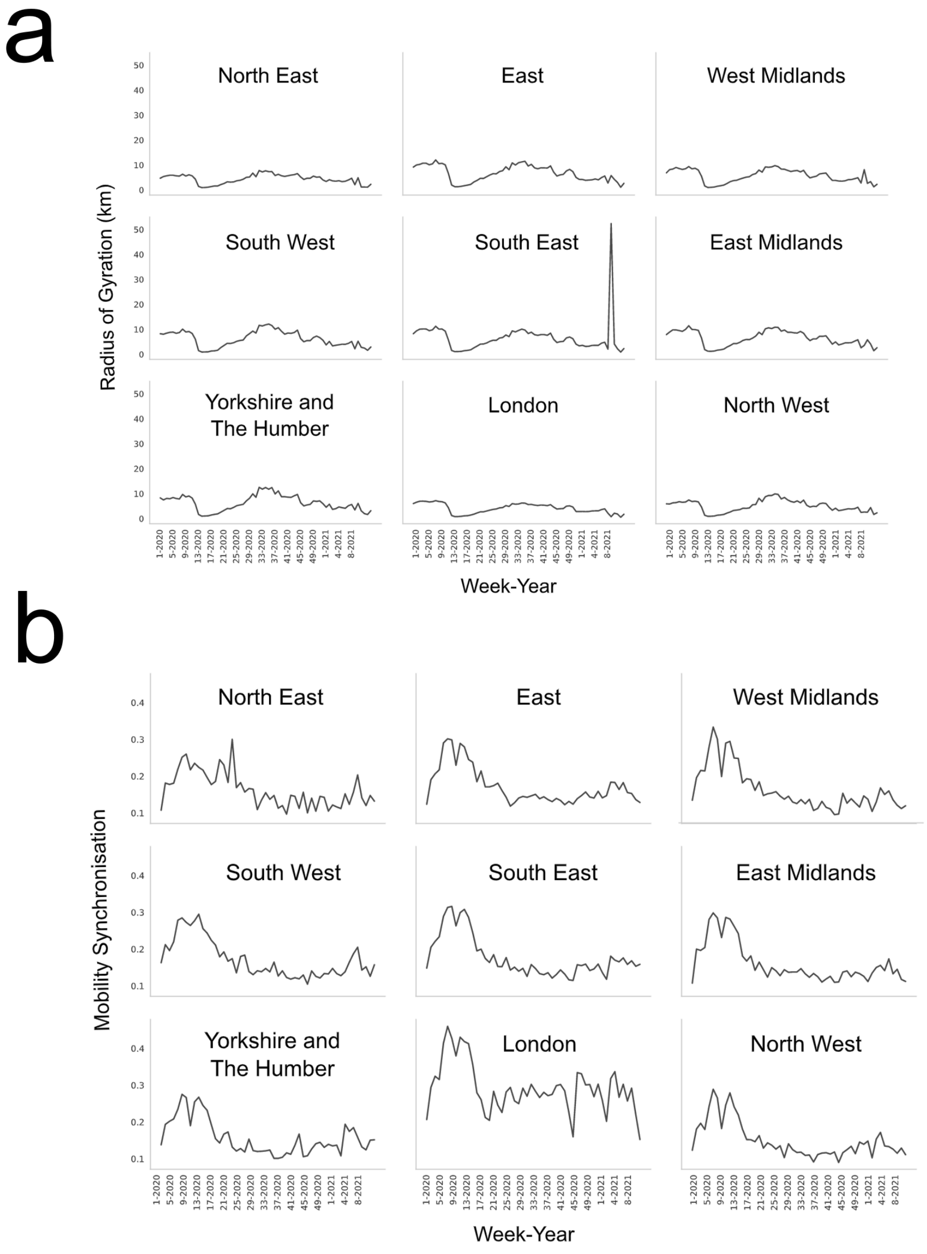

Supplementary Figure 6: Radius of gyration and mobility synchronisation time series for the regions in England. Period from week 1 2020 until week 8 2021.

S5 Mobility Patterns in Northern Ireland

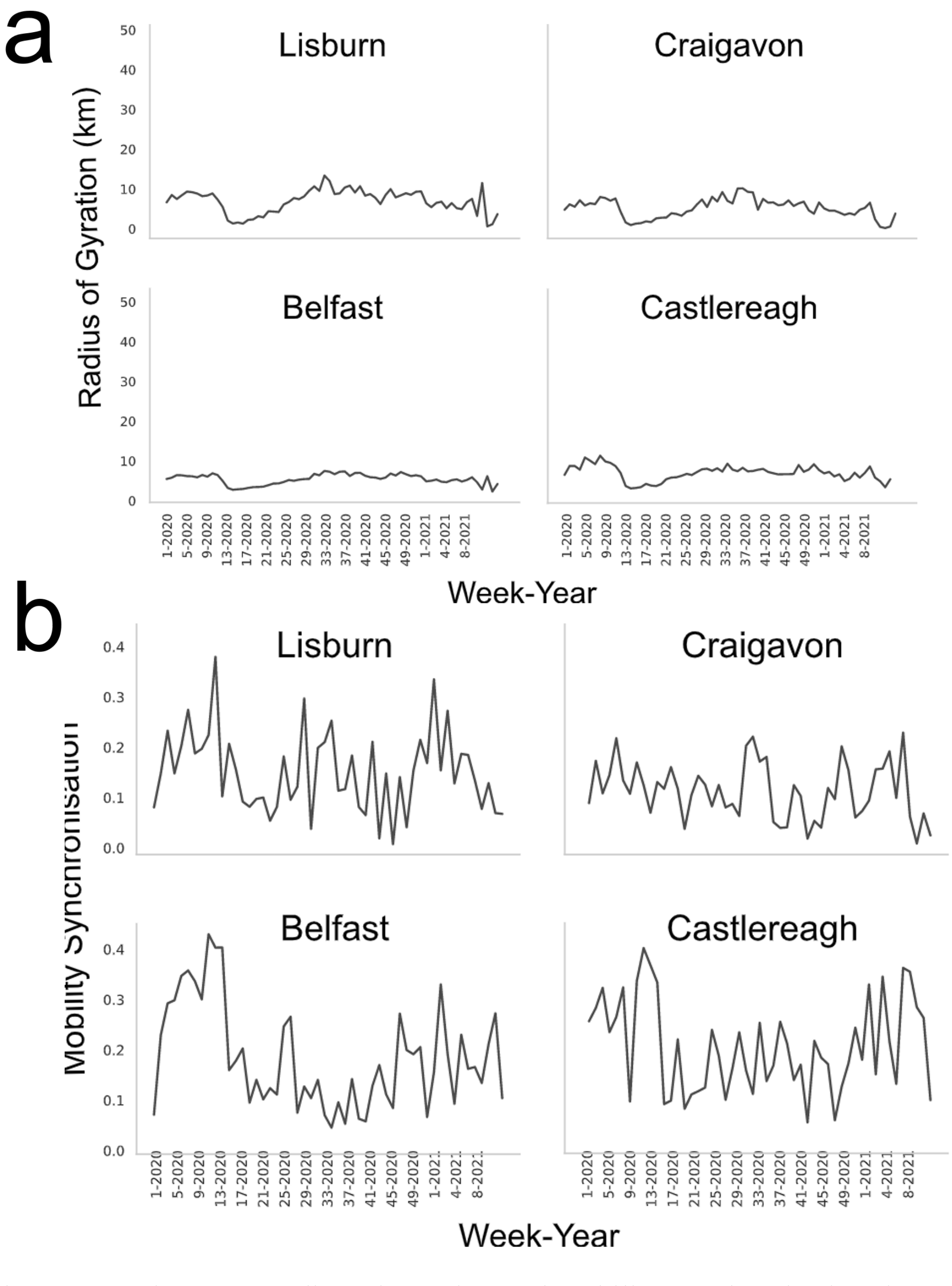

Supplementary Figure 7: Radius of gyration and mobility synchronisation time series for the studied areas in Northern Ireland. Period from week 1 2020 until week 8 2021.

S6 Mobility Patterns in Scotland

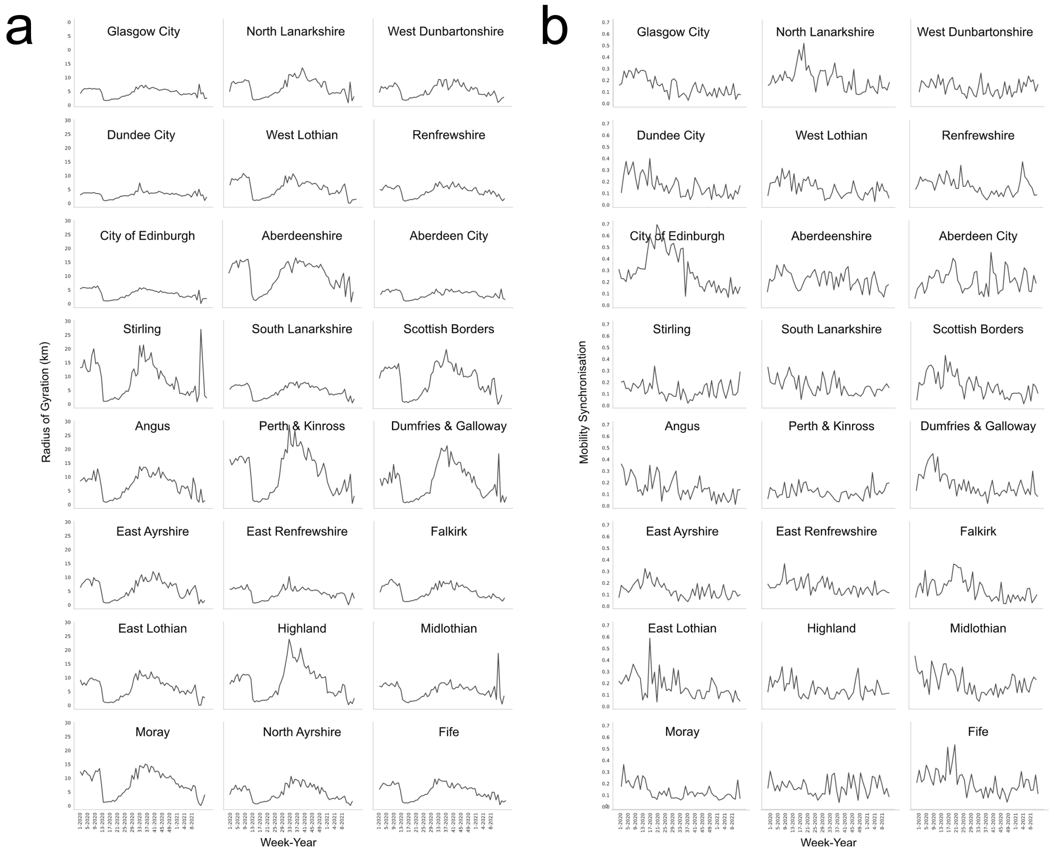

Supplementary Figure 8: Radius of gyration and mobility synchronisation time series for the studied areas in Scotland. Period from week 1 2020 until week 8 2021.

S7 Mobility Patterns in Wales

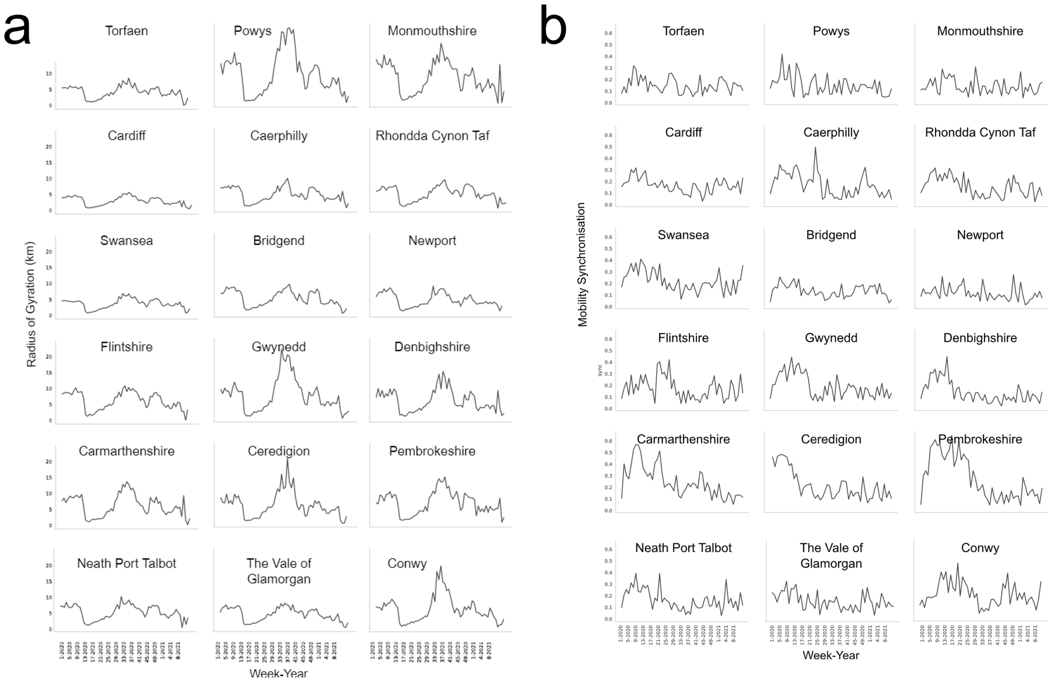

Supplementary Figure 9: Radius of gyration and mobility synchronisation time series for the studied areas in Wales. Period from week 1 2020 until week 8 2021.
