# Supplementary material for: COVID-19 is linked to changes in the time–space dimension of human mobility
Source: Nat Hum Behav. 2023 Jul 27;7(10):1729–39. doi: 10.1038/s41562-023-01660-3 (PMC10593607; doi:10.1038/s41562-023-01660-3)
Supplement: Supplementary file 2 — Reporting Summary [file 41562_2023_1660_MOESM2_ESM.pdf]

nature portfolio

Corresponding author(s): Riccardo Di Clemente

Last updated by author(s): May 31, 2023

# Reporting Summary

Nature Portfolio wishes to improve the reproducibility of the work that we publish. This form provides structure for consistency and transparency in reporting. For further information on Nature Portfolio policies, see our Editorial Policies and the Editorial Policy Checklist.

## Statistics

For all statistical analyses, confirm that the following items are present in the figure legend, table legend, main text, or Methods section.

| n/a | Confirmed | |
|---|---|---|
| ☐ | ☒ | The exact sample size (*n*) for each experimental group/condition, given as a discrete number and unit of measurement |
| ☐ | ☒ | A statement on whether measurements were taken from distinct samples or whether the same sample was measured repeatedly |
| ☒ | ☐ | The statistical test(s) used AND whether they are one- or two-sided<br>*Only common tests should be described solely by name; describe more complex techniques in the Methods section.* |
| ☐ | ☒ | A description of all covariates tested |
| ☐ | ☒ | A description of any assumptions or corrections, such as tests of normality and adjustment for multiple comparisons |
| ☐ | ☒ | A full description of the statistical parameters including central tendency (e.g. means) or other basic estimates (e.g. regression coefficient) AND variation (e.g. standard deviation) or associated estimates of uncertainty (e.g. confidence intervals) |
| ☒ | ☐ | For null hypothesis testing, the test statistic (e.g. *F*, *t*, *r*) with confidence intervals, effect sizes, degrees of freedom and *P* value noted<br>*Give P values as exact values whenever suitable.* |
| ☒ | ☐ | For Bayesian analysis, information on the choice of priors and Markov chain Monte Carlo settings |
| ☒ | ☐ | For hierarchical and complex designs, identification of the appropriate level for tests and full reporting of outcomes |
| ☒ | ☐ | Estimates of effect sizes (e.g. Cohen's *d*, Pearson's *r*), indicating how they were calculated |

*Our web collection on statistics for biologists contains articles on many of the points above.*

## Software and code

Policy information about availability of computer code

| | |
|---|---|
| Data collection | Spectus Inc provided the data used in this research. No additional softer fer data collection was employed. |
| Data analysis | All software used and implemented for the analysis are free and open source. They will be available in the project GitHub page after the publication (covid-uk-mobility.github.io). This information is described in the code availability section of the main manuscript. |

For manuscripts utilizing custom algorithms or software that are central to the research but not yet described in published literature, software must be made available to editors and reviewers. We strongly encourage code deposition in a community repository (e.g. GitHub). See the Nature Portfolio guidelines for submitting code & software for further information.

## Data

Policy information about availability of data

All manuscripts must include a data availability statement. This statement should provide the following information, where applicable:

- Accession codes, unique identifiers, or web links for publicly available datasets
- A description of any restrictions on data availability
- For clinical datasets or third party data, please ensure that the statement adheres to our policy

The dataset with human mobility information was collected and provided by Spectus Ins, and for contractual reasons, it can not be published. The income study employed data from the report on income estimates for small areas in England in 2018 provided by the Office for National Statistics (ONS). Similarly, the unemployment claimant rate analysis used the data published by the ONS, publicly available under the terms of the Open Government Licence. For the green area study, we used the Ordnance Survey Open Greenspace dataset to obtain information on the locations of public parks, playing fields, sports facilities and play areas (OS Open Greenspace). Lastly, the local authorities' income and other socioeconomic indicators were based on data from the UK Census of 2011, which is available for download at the InFuse platform under the terms of the Open Government Licence.

# Field-specific reporting

Please select the one below that is the best fit for your research. If you are not sure, read the appropriate sections before making your selection.

☐ Life sciences ☒ Behavioural & social sciences ☐ Ecological, evolutionary & environmental sciences

For a reference copy of the document with all sections, see nature.com/documents/nr-reporting-summary-flat.pdf

# Behavioural & social sciences study design

All studies must disclose on these points even when the disclosure is negative.

| | |
|---|---|
| Study description | This is a quantitative study based on quantitative data sources. Both the dataset with the human mobility information and the other data sources (e.g. census, ONS and Ordnance Survey data) are composed of quantitative aspects of the population studied. We used location-based data from de-identified mobile phone users (17.8 billion out-of-home trips and about 1 billion radius of gyration records) from January 2019 to early March 2021 to capture the differences in space-time mobility patterns. We observed that a decrease in spatial mobility (gauged with the radius of gyration) is interwoven with the emergence of asynchronous mobility dynamics (computed using the mobility synchronisation metrics) during lockdown periods. Coupling these results with quantitative census data, we noticed that in less urbanised and low-income areas, the spatial mobility dimension had a more significant disruption compared to the urbanised and high-income areas. In contrast, the opposite situation was observed in the temporal dimension. |
| Research sample | The sample studied is composed of mobile phone users in the UK who opted-in to share their information. The UK sample was chosen due to the need for studies concerning human mobility patterns in its population during the COVID-19 pandemic, the availability of the dataset with records of mobility patterns and additional socioeconomics/demographic indications. Our sample comprised about 1 billion users' radius of gyration records and more than 17.8 billion out-of-home trips. The human mobility data was provided by Spectus Inc and was aggregated by the local authority level due to privacy concerns. The radius of gyration data was measured weekly, while the trips were recorded daily. The Census, ONS and Ordnance survey datasets are publically available online and were also aggregated at the local authority level. we conducted experiments to ensure that our sample of the population was representative of the local authorities' total population, as can be seen in the SI information of the paper. |
| Sampling strategy | For all the experiments, we used all UK users in the dataset provided by Spectus Inc. Since these users are associated with a geographic area, they could be mapped to a local authority based on their estimated home location (this procedure is explained in trials in our paper). For the green areas, urbanisation and socioeconomics analyses, we limited our study to the local authorities in England to guarantee the consistency of the data (the same type of data was not available in all four UK countries or was collected using different methodologies.). We performed verifications to ensure that our user base was representative of the population of the local authorities, and the results are illustrated in the supplementary information. |
| Data collection | The human mobility data were collected from anonymous mobile phone users who have opted-in to give access to their location data through a GDPR-compliant framework. This collection was made automatically by an application which sends the data to the company's servers, from where researchers could query the data through an auditable, cloud-hosted sandbox environment. The platform outputs only aggerated information. The remainder dataset was collected from the Office for National Statistics website (income and urbanisation data), Ordnance Survey (green areas data), and InFuse platform (census 2011 data). |
| Timing | From January 2019 to March 2021 |
| Data exclusions | No data was excluded from the analyses. |
| Non-participation | The human mobility data users that removed their location data permission were automatically removed from the sandbox environment. Due to contractual reasons, we can not disclose that exact number. However, we conducted experiments to ensure that our sample of the population was representative of the local authorities' total population, as can be seen in the SI information of the paper. For the census data, there were 31 million men and 32.2 million women in the UK. The estimated populations of the four constituent countries of the UK are 53 million people in England, 5.3 million in Scotland, 3.1 million in Wales and 1.8 million in Northern Ireland. |
| Randomization | Due to the nature of the studies conducted, randomisation was optional. The uses were assigned to a local authority based on their home location. The classification of the local authorities according to their urbanisation level and income/unemployment was based on data published by the Office for National Statistics. The visits to green spaces were gauged based on the location records of the user's device. Lastly, the analyses of the spatial-temporal patterns of human mobility were based on the location records and did not require user randomisation. |

# Reporting for specific materials, systems and methods

We require information from authors about some types of materials, experimental systems and methods used in many studies. Here, indicate whether each material, system or method listed is relevant to your study. If you are not sure if a list item applies to your research, read the appropriate section before selecting a response.

## Materials & experimental systems

| n/a | Involved in the study | |
| --- | --- | --- |
| ☒ | ☐ | Antibodies |
| ☒ | ☐ | Eukaryotic cell lines |
| ☒ | ☐ | Palaeontology and archaeology |
| ☒ | ☐ | Animals and other organisms |
| ☒ | ☐ | Human research participants |
| ☒ | ☐ | Clinical data |
| ☒ | ☐ | Dual use research of concern |

## Methods

| n/a | Involved in the study | |
| --- | --- | --- |
| ☒ | ☐ | ChIP-seq |
| ☒ | ☐ | Flow cytometry |
| ☒ | ☐ | MRI-based neuroimaging |
